# Hepatitis C Among First Nations Australians: A Rethink Is Required to Meet the 2030 Elimination Target

**DOI:** 10.5694/mja2.70299

**Published:** 2026-09-22

**Authors:** James Ward, Emily Pegler, Kellie Stacy, Stephen Harfield

**Affiliations:** ^1^ Poche Centre for Indigenous Health University of Queensland Brisbane Queensland Australia

**Keywords:** Aboriginal and Torres Strait Islander, equity, hepatitis C, Indigenous health

## Abstract

Australia aims to eliminate hepatitis C as a public health threat by 2030 among all populations. It is timely to review national elimination efforts for First Nations Australians and discuss what is required to reach elimination targets. In 2020, First Nations Australians accounted for 18% of people living with chronic hepatitis C, despite representing 4% of the population. Over the past 5 years, about 15% of hepatitis C cases notified nationally occurred among First Nations peoples, with slower uptake of curative direct‐acting antiviral treatment than non‐Indigenous people. Achieving elimination will require implementation of strategies with an equity framework applied to address gaps in data and care cascades for hepatitis C.

## 1 Positionality Statement

We are proud Aboriginal people James Ward (Pitjantjatjara and Narungga), Kellie Stacy (Wiradjuri), Stephen Harfield (Narungga and Ngarrindjeri) and Emily Pegler (Yamatji), who work together at the University of Queensland's Poche Centre for Indigenous Health in Meanjin (Brisbane) and hold cultural connections across South Australia, New South Wales, Western Australia and the Northern Territory. At the Poche Centre, we are committed to undertaking community‐led research that strengthens our understanding of the diverse health priorities of our communities while contributing to improving health and wellbeing outcomes. Our work is grounded in a collective commitment to relational accountability, Aboriginal ways of knowing, being and doing and Indigenous research methods and methodologies. Our diverse identities, lived experiences and values are strengths that guide us in approaching our work with humility, and we continually reflect on how our biases and perspectives influence the ways we engage with communities and interpret knowledge. We acknowledge First Nations peoples with lived and living experience of viral hepatitis as knowledge holders and recognise the wider communities that support them, including service providers and peers whose passion and care help to create safe spaces where people feel seen and respected.

The CONSIDER reporting criteria checklist for health research involving Indigenous peoples was completed for this article and can be found in Table S1 [1].

## 2 Introduction

Australia is a signatory to the World Health Organization (WHO) goal of eliminating hepatitis C virus (HCV) infection as a public health threat by 2030 [2]. To achieve this goal, at least 90% of individuals living with HCV need to be diagnosed and more than 80% need to be treated [3]. In 2024, the global report assessing hepatitis C elimination in 33 countries shows that Australia has achieved the 2025 targets for diagnosing and treating people with HCV, indicating significant progress towards the 2030 elimination target [4]. Australia's elimination strategies encompass all populations, including First Nations peoples identified as a priority population. Australia's overall progress, however, masks inequities for First Nations peoples, who continue to experience diagnosis rates and morbidity due to untreated HCV infection, including hepatocellular carcinoma, and a substantially lower survival rate than non‐Indigenous peoples [5]. This article outlines strategies for First Nations peoples to ensure this population is not left behind at or in 2030.

An anchor in the strategy to achieve elimination of hepatitis C in Australia was the introduction of curative direct‐acting agents (DAAs) that became available on the Australian Pharmaceutical Benefits Scheme a decade ago (March 2016) [4]. These DAAs are highly efficacious, curing HCV infection in over 98% of patients, with relatively short treatment cycles (4–12 weeks) and minimal adverse effects. Since their introduction, there have been great efforts to ensure DAAs are widely available through a variety of clinical settings such as primary health care, drug and alcohol services and in Australian prisons [6, 7].

In the decade since DAAs were introduced, nationally, the total number of all people living with HCV and the incidence of HCV infection among prisoners and among people who inject drugs have almost halved [8]. However, for First Nations peoples, who comprise 4% of the Australian population, the impact has been minimal. In 2020, an estimated 21,000 First Nations peoples were living with chronic HCV infection, representing 18% of the estimated population living with HCV in Australia [9]. Between 2016 and 2024, annual HCV infection notifications decreased by 41% in the non‐Indigenous population and by 4% among First Nations peoples [10], but in recent years, notifications have been higher than in 2016 [10, 11]. In 2024, the age‐standardised HCV infection notification rate among First Nations peoples was more than seven times that of non‐Indigenous peoples (165 vs. 23 per 100,000 population, respectively), and the notification rate has been consistently 10‐fold higher among First Nations peoples aged 15–24 in the past decade than their non‐Indigenous counterparts [11]. Given that DAAs are a primary strategy to achieve elimination in the Australian context [12], and in the absence of treatment data by Indigenous status at a truly national level, there is reliance on sentinel studies from primary health care to understand DAA uptake among First Nations peoples. These studies arise from primary care [13, 14], prisons [15, 16], needle syringe programmes and drug and alcohol services [17, 18]. In most of these studies, treatment uptake is reported to be lower among First Nations than non‐Indigenous people [9, 19], and loss to follow‐up [14, 20] and re‐infection post‐treatment, especially in prisons [15, 16], are significant barriers to achieving elimination. Despite the availability of two major sentinel surveillance services, including ACCESS and ATLAS, these systems represent a small proportion of potential services that could provide hepatitis C testing and treatment to First Nations peoples [21, 22]. Therefore, efforts are required to improve national coordination related to both testing and treatment of HCV infection to monitor progress towards elimination.

We outline four strategies to ensure First Nations peoples are not left behind in Australia's commitment to eliminating hepatitis C by 2030.

## 3 Strategy 1: Enhanced Epidemiological Data

Given the complexity and intersectionality of issues impacting First Nations peoples at risk of and living with HCV, it is fundamental to have a detailed understanding of HCV epidemiology for this population to shape strategies moving forward. To achieve elimination, 90% of people with HCV infection should be diagnosed, and 80% of people living with HCV must be treated and achieve a sustained virologic response. Addressing hepatitis C data gaps should be a core priority of the newly created Australian Centre for Disease Control.

### 3.1 Improving HCV Infection Notification and Testing Data

Priority should be given to improving the completeness of First Nations status in HCV notification data. Currently, New South Wales and Victoria have < 50% of all diagnoses reported with First Nations status. Efforts are required to improve the completeness and accuracy of these two jurisdictions' data systems, in alignment with other Australian jurisdictions.

Further, more granular data, which include context of testing (prisons, Aboriginal Community Controlled Health Services [ACCHS] and community clinics), would help elimination efforts. Similarly, aligning RNA testing (both positive and negative) with national notification data will assist efforts to identify whether high or low notification rates in a region reflect testing rates.

### 3.2 Improving HCV Infection Treatment Data

Key questions remain about treatment and outcomes among First Nations peoples. National targets are required to reduce the number of First Nations people living with HCV. National data on treatment occurrences and re‐infection should be enumerated for First Nations peoples and by settings (primary care, prisons, Alcohol and Other Drug services and harm reduction services) and, where possible, at jurisdictional and national levels. Understanding reinfection and retreatment is also essential to guide effective elimination strategies for First Nations peoples.

### 3.3 Enhanced Drug Use Data

Current drug use data among First Nations peoples, we believe, falls short of telling a complete story about drug use, particularly injecting drug use in First Nations communities nationally. One example has been the changing nature of drug use in the past decade, from opiates to more amphetamine‐based drugs, and reported and apparent increases in injecting behaviour across all regions. As such, it would be beneficial to have an Indigenous‐led longer‐term surveillance system that monitors drug use, including injecting drug use in First Nations communities. Current methods for collecting information about drug use are falling short in addressing knowledge of drug use and injecting drug use in our communities.

## 4 Strategy 2: Harm Reduction

Given there has been no notable decrease in incident cases of HCV infection among First Nations peoples since 2016, we propose a substantial expansion of culturally safe and accessible harm reduction programmes to increase coverage per injection with clean injecting equipment, especially in ACCHS and in Australian prisons, to reduce incidence. In addition, this would assist in reducing levels of receptive sharing of drug‐injecting equipment among First Nations peoples, which is reported at almost double that of non‐Indigenous Australians [23].

Increasing drug treatment and rehabilitation programmes, including improved coverage of opioid agonist therapy, and other drug treatment programmes specific to First Nations peoples, is also required, including programmes designed and delivered by ACCHS [24].

Optimising HCV prevention strategies as well as management in prisons will make a major contribution towards Australia's efforts to eliminate HCV infection as a public health threat by 2030 [7]. Streamlined treatment pathways that encompass prison transfers are required [25, 26], and the implementation and evaluation of regulated prison needle syringe programmes are required to reduce HCV incidence and reinfection after treatment, which is higher among First Nations prisoners.

## 5 Strategy 3. Addressing the Social and Cultural Determinants of Injecting Drug Use

Social determinants of health include access to care, housing, employment and education. Gaps exist in knowledge of how HCV is transmitted and in curative drugs. Knowledge of HCV transmission was low in First Nations adolescent offenders in New South Wales [7], with over 50% not knowing how it is transmitted and fewer than 10% being able to identify sharing needles as a risk.

Financial incentives to reduce drop‐off in the testing and treatment cascade would facilitate progress towards hepatitis C elimination in Australia. Programmes such as the Motivate C and Deadly Liver Mob projects have investigated favourably whether cash rewards encourage people with HCV infection to seek treatment from community treatment providers [27, 28].

Addressing the determinants of injecting drug use must be central to elimination efforts. Programmes that are packaged to manage underlying issues such as trauma and intergenerational trauma are required. First Nations people with HCV infection have a high prevalence of depression (72%) and anxiety (61%) [29]. On the other hand, people diagnosed with mental illness are more likely to have risk factors for HCV transmission, such as unsafe sexual behaviours [30], and the prevalence of HCV infection is high in this population, with 11% among patients admitted urgently to psychiatric inpatient facilities [31].

## 6 Strategy 4. Improving All Aspects of the Testing and Treatment Cascade

To improve access to care, it will be necessary to consider flexible and consistent outreach programmes and the employment of peers within existing services to deal with issues in the access, testing and treatment pathway [32]. Peer workers can help people at risk of or affected by HCV infection navigate complex health systems, increase treatment knowledge, reduce loss to follow‐up, improve treatment completion and mitigate stigma, discrimination and shame associated with HCV infection [33].

Rapid point‐of‐care testing platforms approved for use in Australia should increase testing options for First Nations people. However, the uptake of HCV point‐of‐care testing remains limited within ACCHS despite their widespread use for respiratory diseases and sexually transmissible infections. As a result, the benefits of rapid testing are not yet reaching many communities that could benefit most, highlighting a substantial gap in equitable access and implementation. In scaling up these diagnostics, it will be important to consider their placement in settings where incidence and prevalence are greatest, and the provision of ongoing training and ensuring the capacity of services to make the most of these tests. In addition, high‐coverage testing protocols, simplified assessment protocols and streamlined DAA treatment pathways require further implementation to achieve elimination.

## 7 Conclusion

Eliminating hepatitis C as a public health threat in Australia is predicted to be on track, but not for First Nations peoples. Elimination requires coordinated, multilevel, multisectoral, place‐focussed and evidence‐based interventions that address the social and cultural determinants of HCV infection across individuals, communities, health services and national policy. Indigenous leadership and partnerships are necessary pre‐conditions for the elimination of viral hepatitis. As is resourcing that enables decolonised responses that centre First Nations health knowledges, and care that is holistic, person‐centred and community‐enabled and prioritised. Our approach must be with a focus on equity and potency to ensure no one group is left behind in Australia when achieving elimination of HCV infection.

## Author Contributions

**James Ward:** conceptualisation, writing (original draft), writing (review and editing). **Emily Pegler:** writing (review and editing). **Kellie Stacy:** writing (review and editing). **Stephen Harfield:** writing (review and editing).

## Funding

The authors have nothing to report.

## Disclosure

Not commissioned; externally peer reviewed.

## Conflicts of Interest

The authors declare no conflicts of interest.

## Supporting information


**Table S1.** CONSIDER statement.

## Data Availability

The authors have nothing to report.
