# Supplementary material for: Hepatitis C Among First Nations Australians: A Rethink Is Required to Meet the 2030 Elimination Target
Source: Med J Aust. 2026 Sep 22;224(9):e70299. doi: 10.5694/mja2.70299 (PMC13597174; doi:10.5694/mja2.70299)
Supplement: Supplementary file 1 [file MJA2-224-0-s001.pdf]

## Supporting Information

## Supplementary material

**This appendix was part of the submitted manuscript and has been peer reviewed. It is posted as supplied by the authors.**

Appendix to: J. Ward, E. Pegler, K. Stacy and S. Harfield. Hepatitis C among First Nations Australians: A Rethink Is Required to Meet the 2030 Elimination Target. *Medical Journal of Australia* 2026; doi: 10.5694/mja2.70299.

**CONSIDER Statement**

Guest Editors of the 2026 *Indigenous Health Special Collection* acknowledge the Indigenous expertise that informed the establishment of the CONSolIDated critERtia for strengthening the reporting of health research involving Indigenous Peoples (CONSIDER) statement.

Authors should indicate how they have supported ethical publishing and reporting practices by providing the details of the research practices aligned with this publication in accordance with the CONSIDER statement. The reporting should not exceed two pages. This reporting will be published as online supplementary information. Detailed items can be accessed in the publication:

https://bmcmedresmethodol.biomedcentral.com/articles/10.1186/s12874-019-0815-8

**Table S1. CONSIDER Statement.**

| **Governance** |
|---|
| This perspective highlights inequities in hepatitis C elimination among Aboriginal and Torres Strait Islander peoples and proposes four strategies to accelerate progress towards elimination. All authors are Aboriginal researchers whose expertise informed the development of this manuscript. J.W. has over 25 years of experience in Aboriginal public health policy across government and non-government and has established a nationally recognised program of research focused on the epidemiology and prevention of STIs and BBVs among Aboriginal and Torres Strait Islander peoples. E.P. and S.H. are early career researchers whose expertise spans Indigenous health, viral hepatitis and epidemiology K.S. has extensive expertise in sexual health and community-led research, with a strong focus on culturally safe and community-prioritised approaches. Development of this manuscript was guided by the collective expertise of the author team. Decisions regarding the framing, interpretation of evidence and proposed strategies were made collaboratively to ensure the paper reflects a strengths-based approach, centres Aboriginal and Torres Strait Islander perspectives, and promotes community-led, feasible, and culturally responsive solutions for hepatitis C elimination. |
| **Prioritization** |
| This perspective piece highlights persistent inequities in hepatitis C among Aboriginal and Torres Strait Islander peoples and supports Australia's commitment to eliminating hepatitis C as a public health threat by 2030. It outlines the need for coordinated, multilevel, and cross-sector strategies that address social and cultural determinants, improve equitable access to prevention, testing, and treatment, and strengthen data collection and surveillance. The priorities outlined are informed by the authors' extensive experience in Aboriginal and Torres Strait Islander health and viral hepatitis research. E.P.'s recent research on drug harm reduction services across metropolitan and regional Queensland identified the need for culturally safe, accessible harm reduction services and peer-led prison transition programs to support continuity of hepatitis C care following release from custody. These findings, alongside the collective expertise of the author team, informed the strategies proposed to advance equitable, community-led hepatitis C elimination efforts. |
| **Relationships (Indigenous stakeholders/participants and Research Team** |
| This manuscript did not involve primary research or the recruitment of Indigenous participants; therefore, formal ethics approval was not required. The manuscript was developed by an Aboriginal-led author team with longstanding experience working in partnership with Aboriginal and Torres Strait Islander communities, Aboriginal Community Controlled Health Organisations, policymakers and health services. The perspectives and recommendations presented are informed by these established relationships, the authors' collective expertise in Indigenous health and viral hepatitis, and a shared commitment to Indigenous self-determination, culturally safe practice and community-led approaches to hepatitis C elimination. |

| **Methodologies** |
| --- |
| This manuscript did not involve primary research or the recruitment of participants; therefore, formal ethics approval was not required. All authors critically considered the framing of the manuscript to avoid attributing responsibility for hepatitis C inequities on Aboriginal and Torres Strait Islander peoples. Instead, the paper acknowledges the impacts of colonisation, racism and structural inequities, and advocates for culturally safe, community-led solutions. |
| **Participation** |
| This manuscript did not involve primary research, data collection, or recruitment of Indigenous participants. The authors drew on contemporary peer-reviewed evidence and national surveillance data, including Aboriginal and Torres Strait Islander-led research and reports where available. Key sources included the systematic review and meta-analysis led by Narungga researcher Dr Simon Graham: *Prevalence of Hepatitis C Among Australian Aboriginal and Torres Strait Islander People: A Systematic Review and Meta-Analysis* (Hepatology Monthly, 2016;16(7)). |
| **Capacity** |
| The development of this manuscript provided an opportunity to support capacity strengthening among the author team, particularly early career researchers E.P. and S.H. Through collaborative authorship and mentorship, the manuscript has contributed to developing research, writing and publication skills. This reflects the CONSIDER Statement's emphasis on building Indigenous research capacity through meaningful collaboration, mentorship, and knowledge exchange. |
| **Analysis and interpretation** |
| All authors collectively interpreted and framed this perspective through Aboriginal and Torres Strait Islander ways of knowing, being and doing. Drawing on their collective expertise, positionality and professional experience, authors ensured the manuscript adopted a strengths-based approach, avoided deficit-based narratives, and emphasised the structural and systemic factors contributing to hepatitis C inequities. |
| **Dissemination** |
| This perspective piece is intended for national and international readership and aims to contribute to ongoing work addressing hepatitis C inequities. Publication in a peer-reviewed journal, particularly within the special NAIDOC Week issue, is important for recognising and amplifying the expertise of Aboriginal and Torres Strait Islander peoples working in academia. All authors are affiliated with the UQ Poche Centre for Indigenous Health in Meanjin (Brisbane). Following publication it will be shared through professional networks social media (including LinkedIn) to support broader dissemination and impact. |
